# How do some nurses achieve post-traumatic growth in stressful situations? Analysis of the role of hope, meaning of life, and resilience with the mediating role of coping self-efficacy

**DOI:** 10.1016/j.heliyon.2024.e40038

**Published:** 2024-11-01

**Authors:** Majid Yousefi Afrashteh, Masoumeh Moradi, Leila Rahmandel

**Affiliations:** Department of Psychology, Faculty of Humanities, University of Zanjan, Zanjan, Iran

**Keywords:** Meaning of life, Post-traumatic growth, Coping self-efficacy, Nurses

## Abstract

**Background:**

This study aimed to investigate the relationship between hope, the meaning of life, resilience, and post-traumatic growth (PTG), with coping self-efficacy as a mediating factor among nurses working in COVID-19 care departments.

**Methods:**

This was a quantitative cross-sectional study involving 263 nurses from three university hospitals in Zanjan, Iran, in 2022. Data were collected using Connor and Davidson's Resilience Questionnaire, Schneider's Hope scale, Steger's Meaning in Life questionnaire, Tedeschi and Calhoun's PTG inventory, and Chesney's coping self-efficacy scale. Data were analyzed using SPSS-26 and LISREL-10.2 software.

**Results:**

Path analysis was used to analyze the causal model, which demonstrated a good fit with the data., the results showed. The results revealed direct and significant effects of resilience (ƿ<0.05, β = 0.14), hope (ƿ<0.05), β = 0.16), the meaning of life (ƿ<0.05, β = 0.13), and coping self-efficacy (ƿ<0.05, β = 0.20) on PTG among nurses. Additionally, the mediation of coping self-efficacy in the relationship between resilience, hope, meaning of life, and PTG in nurses was confirmed through the analysis of multiple mediators.

**Conclusion:**

The study revealed that the combination of resilience, hope, and the meaning of life, mediated by coping self-efficacy, has significant predictive power for PTG in nurses.

## 1 Background

The global spread of COVID-19 adversely affected everyone [1] including nurses who are more exposed to many occupational stresses compared to others due to their high workload, distance from family, work shifts, and fear of getting infected with the virus, among other factors [2,3]. The health of nurses is endangered by work stress [4,5] including their mental health [6,7] However, delivering medical care in demanding and critical circumstances, such as during the COVID-19 pandemic, does not always lead to negative changes in nurses but can also have favorable effects [8,9]. Post-traumatic growth (PTG) refers to the beneficial psychological changes that a person experiences as a result of coping mechanisms when confronted with significant life events, crises, or traumatic events [10]. PTG involves a sublime understanding of life, one's self, and ideals, leading to improved psychological development, empathy, bettering interpersonal relationships, and reevaluating priorities in life [11]. Nurses engaged in clinical work may benefit professionally and personally from PTG, which also improves their capacity for stress management [12].

Resilience is among the factors that can influence recovery from a traumatic event [13]. Resilience can be defined as a person's capacity to deal with adversity, resistance to illness, and adaptability to changing circumstances to preserve mental health [14,15]. The overall framework of resilience is a dynamic process in which a person exhibits positive adaptive behaviors when facing catastrophic adversities [16], which leads to individual stability under challenging circumstances [17]. According to studies, resilience is a key factor in PTG [18,19]. People with higher resilience are more likely to achieve positive meaning during stressful experiences, and they effectively face their life challenges, reduce the negative effects of the traumatic event, and adapt themselves to life stressors [20]. In Shaffer and Moose's model, resilience is one of the positive outcomes that arise from afterlife crises and is the strongest predictor of PTG [21].

Hope is another concept associated with PTG. Additionally, as an emotional process, hope increases people's adaptability, and helps them cope with problems [22] leading to improved psychological health. This suggests that coping with a stressful situation is easier when there is hope, as it increases the chances of a positive outcome. High expectations for the outcome will lead to better performance under pressure. High levels of hope may make people more willing and motivated to deal with stressful situations because they perceive stressors as challenging (rather than threatening) [23]. Hope boosts nurses' self-efficacy and helps them deal with difficult situations [24]. Numerous studies have confirmed the positive association between hope and resilience [25], and hope and growth [26].

Meaning of life is another important construct for PTG [27]. Coherence, having a purpose in life, pursuing worthwhile goals, and accomplishing those goals with a sense of satisfaction define a meaningful life [28]. More precisely, the meaning of life refers to a sense of connection with existence, having a purpose, pursuing values, and striving for growth and perfection [29]. The meaning of life is connected to all aspects of well-being, probably playing an important role in emotional and physical well-being [30]. Meaningfulness of life is a strong predictor of personal life satisfaction and a crucial psychological factor that enhances well-being [31]. The meaning of our experiences becomes more profound through the challenges of critical situations and traumatic events [32]. This factor improves a person's capacity to deal with traumatic events and problems [33]. Having meaning in life is one of the predictors of human well-being and life satisfaction. Studies show that the concept of the meaning of life is closely related to people's psychological health and well-being, and it causes the reduction of negative emotions such as anxiety, depression, and problems after psychological injuries [34]. Meaningfulness of life helps nurses consider life purposeful, cope with the problems of their stressful job, and understand the value of their work as an extraordinary job [35].

The role of coping self-efficacy can be studied as a mediating variable in explaining the relationship between the meaningfulness of life, hope, and resilience with PTG. The ability or self-confidence of a person to cope well with a difficult or traumatic situation is known as coping self-efficacy [36]. Self-efficacy is regarded as a prerequisite for efficient coping, during which a person analyzes his/her capacity to manage a dangerous circumstance, according to Bandura's (1997) self-efficacy thesis [37]. According to Bousmans et al.’s study (2016), psychological recovery following trauma is significantly influenced by coping self-efficacy [38]. Additionally, Ebrin and Delongis's study (1996) demonstrates that, in general, nurses utilize confrontational methods when dealing with professional problems [39]. The positive outcomes of the COVID-19 pandemic's difficult experiences are amplified by a sense of meaning in life, demonstrated through resilience in the form of PTG [35]. According to Liu et al.’s study (2021), strengthening nurses' resilience also boosts their PTG [40]. The results of Lee's study (2021) also demonstrated that the tactics for enhancing hope, PTG, and finding jobs will help boost the resilience of beginner nurses [41].

Since the COVID-19 pandemic over the past few years, Considering the direct and inevitable contact of nurses on the front line of fighting diseases, it seems that this group is a suitable candidate for studying psychological factors effective in maintaining mental health and promoting growth after injury. According to the research background, it is clear that so far the focus of research has been mainly on the negative effects of the coronavirus, and less research has paid attention to investigating the dimensions and potential positive effects in this field. In line with this research gap, the present study studied the related positive psychological variables (resilience, meaningfulness, and hope). It was carried out to predict PTG in nurses in the care department of Corona patients and consider the mediating role of coping self-efficacy. The results of this research can provide a new perspective on the role of positive psychological variables in moderating the impact of difficult situations. The hypotheses of the research are given below.

• Resilience positively and directly affects coping self-efficacy.
• Hope positively and directly coping self-efficacy.
• The meaning of life positively and directly affects coping self-efficacy.
• Resilience positively and directly effects PTG.
• Hope positively and directly effects PTG.
• The meaning of life positively and directly effects PTG.
• Resilience indirectly effects PTG (through coping self-efficacy).
• Hope indirectly effects PTG (through coping self-efficacy).
• Meaning of life indirectly effects PTG (through coping self-efficacy).

**Fig. 1:**
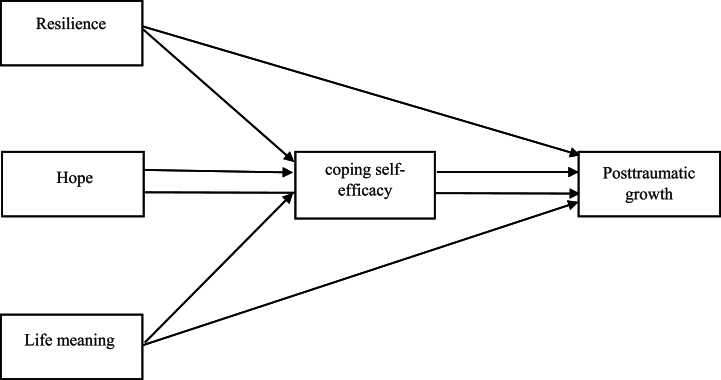
Conceptual model of the research.

Fig. 1 shows the conceptual model of the research that reflects the hypotheses.

### 1.1 Participants and procedure

A cross-sectional study was carried out to have a look at the relationships between resilience, hopefulness, and life meaning with posttraumatic growth among nurses as well as the mediating effects of coping self-efficacy. The participants were nurses in the care department for patients with COVID-19 recruited from three university hospitals located in Zanjan, Iran. Recruitment occurred from April 16th to June 15th, 2022. Inclusion criteria included full-time employment history for two or more years, working in the care of patients with Covid-19 for at least 6 months, understanding the purpose of the study, and informed consent to participate in the study. The criterion for exclusion the research was incomplete answers to the questionnaires. According to the inclusion criteria, 263 nurses participated in the present study, of which 77 (29.28 %) were men and 186 (70.72 %) were womenAccording to Klein's recommendation (42), this number of samples is sufficient to perform path analysis. The Declaration of Helsinki applies the study relative to clinical non-interventional research. Demographic information, including age, education, marital status, and type of therapy are presented in Table 1.

**Table 1:** Demographic statistics of the subjects (n = 263).

Characteristics	M (SD)	N (%)
**Age(Year)**	34.22 (17.38)	
20–25		62 (23.57)
26–30		86 (32.70)
31–35		59 (22.43)
36–40		36(13.69)
41–45		20(07.60)
**Education**		
Associate Degree		64 (24.33)
Bachelor		163 (61.98)
Master		36 (13.69)
**Marital Statue**		
Single		43 (16.35)
Married		220 (83.65)
**Length of Working Experience(Year)**		
1–5		57 (21.67)
6–10		90 (34.22)
11–15		57 (21.67)
16–20		38(14.45)
21–25		21(08.98)

**Fig. 2:**
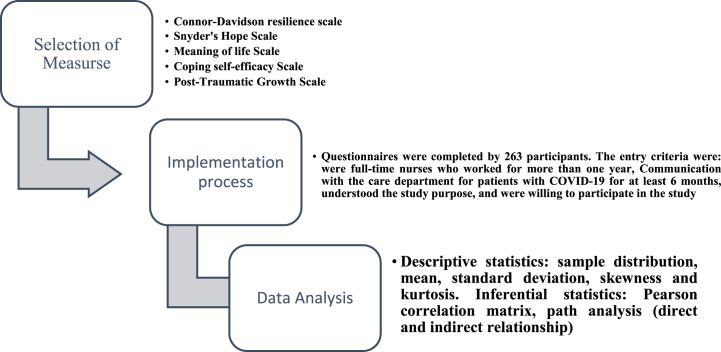
Flowchart of the research method.

Fig. 2 shows the flowchart of the research method.

## 2 Measures

**Connor-Davidson resilience scale (CD-RISC):** This scale was developed in 2003 by Connor and Davidson, which consists of 25 statements and is scored based on a 5-point Likert scale (from 0 for always false to 4 for always true). The score of this questionnaire ranges between zero and 100. This questionnaire consists of 5 subscales of the self-competence image, trust in self-intuition, a positive attitude toward changes and safe relationships, spiritual control, and influences. Connor and Davidson confirmed the test's validity and the reliability coefficient of the test was 0.87(43). In Iran, Hagh Ranjbar et al.’s study (2011) calculated the validity of this questionnaire [42]. The reliability of the test was assessed with Cronbach's alpha which was revealed to be 0.89. The Cronbach's alpha for the reliability of the test was 0.77 in the present study.

**Snyder's Hope Scale(AHS):** This Scale was developed by Schneider et al., in 1991 and consisted of 12 statements scored based on a 4-point Likert scale (from 1 for completely false to 4 for completely true). The score of the questionnaire ranged between 12 and 48. Schneider et al. (1991)(45) reported a Cronbach's alpha of 0.70 for the reliability of the test. According to Khalji's study in Iran, Cronbach's alpha for the reliability of this questionnaire was 0.70, and retesting after one month revealed a reliability coefficient of 0.74 [43]. The Cronbach's alpha for the reliability of the test was 0.86 in the present study.

**Meaning of Life Scale (MLQ):** The questionnaire, developed by Steger et al. (2006), measures the two dimensions of the existence of meaning and the search for meaning. This questionnaire consists of 10 statements and is scored using a 7-point Likert scale ranging from 1 (completely false) to 7 (completely true). The overall score of the test ranges between 10 and 70. In Steger et al.'s study (2006), the validity of the existence of meaning subscales and the search for meaning subscales were 0.86 and 0.87 respectively. Additionally, the reliability of the subscales measuring the presence of meaning and the search for meaning was calculated to be 0.70 and 0.73, respectively [44]. Ishtahad (2009) found that the presence of the meaning subscale had a test-retest reliability of 0.84 in Iran while the search for the meaning subscale had a test-retest reliability of 0.74 [45]. The Cronbach's alpha was reported to be 0.78 for the presence of the meaning subscale and 0.75 for the search for meaning subscale. The Cronbach's alpha for the reliability of the test was 0.82 in the present study.

**Coping Self-Efficacy Scale(CSES):** This 26-item scale was developed by Chesney et al., in 2006, and is scored based on a 3-point Likert scale (zero for cannot do at all, 1 to 5 for Moderately certain can do, and 5 to 10 for Certain can do). Three subscales of this questionnaire included halting negative feelings and thoughts, problem-focused coping, and seeking help from family and friends. According to Chesney et al. (2006), this questionnaire's internal reliability coefficient was 0.91 using Cronbach's alpha method [36]. Using confirmatory component analysis, Bahramian et al. (2015) in Iran reported the validity of this questionnaire [46]. The Cronbach's alpha for the reliability of the test was 0.79 in the present study.

**Post-Traumatic Growth Scale (PTGI):** The 21-item questionnaire was developed by Tedeschi and Calhoun in 1996 and is scored based on a 6-point Likert scale (from zero for not at all to 5 for too much). The score of this test range between 0 and 105. Five subscales are included in this questionnaire: Personal Strength, New Possibilities, Improved Relationships, Spiritual Growth, and Appreciation for Life. Using Cronbach's alpha technique, Tedeschi and Calhoun (2004) reported the reliability of this questionnaire to be 0.96 [9]. According to Seyed Mahmoudi's study (2013) in Iran, Cronbach's alpha method yielded a reliability coefficient of 0.92 [47]. The Cronbach's alpha for the reliability of the test was 0.80 in the present study.

## 3 Results

Table 2 shows descriptive information including mean, standard deviation, and correlation coefficient between variables. for instance, the mean coping self-efficacy is 98.97, with a standard deviation 20.95. All correlation coefficients in Table 1 are statistically significant at a level of P < 0.05. The correlation coefficient of PTG with hope is 0.24, with resilience is 0.24, with the meaning of life is 0.23, and with coping self-efficacy is 0.29. The correlations of resilience, hope, and the meaning of life with coping self-efficacy are 0.23, 0.21, and 0.24, respectively.

**Table 2:** Descriptive information and correlation between variables.

Variables	Mean	SD	1	2	3	4
1-Resilience	68.56	16.68	1			
2-Hope	36.25	7.83	0.13	1		
3-Life meaning	37.59	8.82	0.26	0.15	1	
4-Coping self-efficacy	98.97	20.95	0.23	0.21	0.24	1
5-Posttraumatic growth	53.17	10.15	0.24	0.24	0.23	0.29

The model's goodness of fit was assessed using various indices. The model was found to be saturated, with CFI = 1.000, TLI = 1.000, RMSEA = 0.000, and SRMR = 0.000, indicating a perfect fit of the data to the model. The findings of the path analysis, including standard parameter and t values, are presented in Table 3 for the examination of the relationships between variables.

**Table 3:** Path analysis coefficients for the relationship between variables.

Path	Standard Estimate	t-value	p-value
**Direct**			
Resilience → PTG	0.14	2.31	ƿ<0.05
Hope → PTG	0.16	2.71	ƿ <0.01
Life meaning → PTG	0.13	2.06	ƿ <0.05
CSE → PTG	0.20	3.16	ƿ <0.01
Resilience → CSE	0.17	2.68	ƿ <0.01
Hope → CSE	0.16	2.80	ƿ <0.01
Life meaning → CSE	0.18	2.63	ƿ <0.01
**Indirect**			
Re → CSE→ PTG	0.033	2.04	ƿ <0.05
Ho → CSE→ PTG	0.034	2.09	ƿ <0.05
LM → CSE→ PTG	0.031	2.01	ƿ <0.05
**Total**			ƿ <0.05
Resilience → PTG	0.17	2.82	ƿ <0.01
Hope → PTG	0.19	3.21	ƿ <0.01
Life meaning → PTG	0.16	2.60	ƿ <0.01

PTG: Post-Traumatic Growth, CSE: Coping self-efficacy, Re: Resilience, Ho: Hope, LM: Life meaning.

Based on the results of the path analysis presented in Tables 3 and it became clear that resilience (β = 0.14), hope (β = 0.16), the meaning of life (β = −0.13), and coping self-efficacy (β = 0.20) have a significant direct effect on PTG. Additionally, resilience (β = −0.17), hope (β = −0.16), and the meaning of life (β = 0.18) have a significant relationship with coping self-efficacy. Furthermore, resilience (β = 0.033), hope (β = 0.034), and the meaning of life (β = 0.031) have a significant indirect effect on PTG. Therefore, the results confirm a direct relationship between resilience, hope, and the meaning of life with PTG, as well as the mediating role of coping self-efficacy in this correlation.

**Fig. 3:**
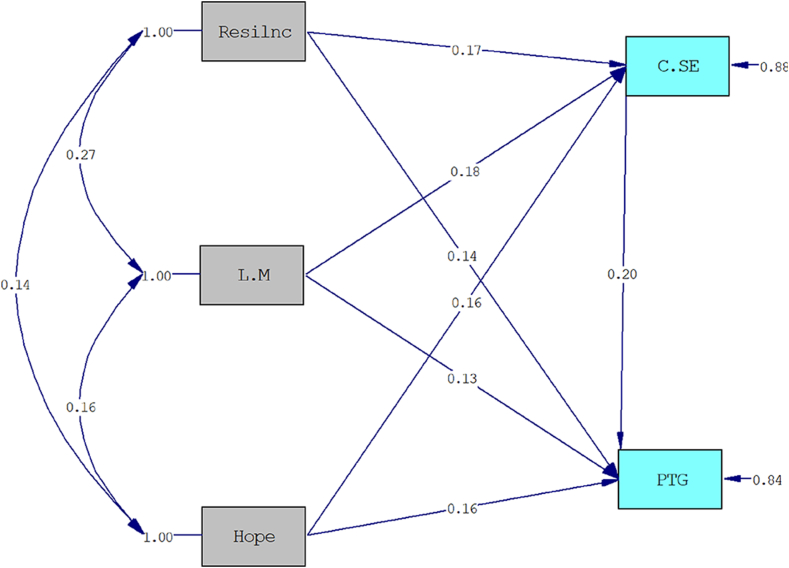
Standard parameter the relationship between variables. Note: Resilnc: Resiliance; L.M: Life Meaning; C.SE: Coping Self-Efficacy; PTG: Post-traumatic Growth.

**Fig. 4:**
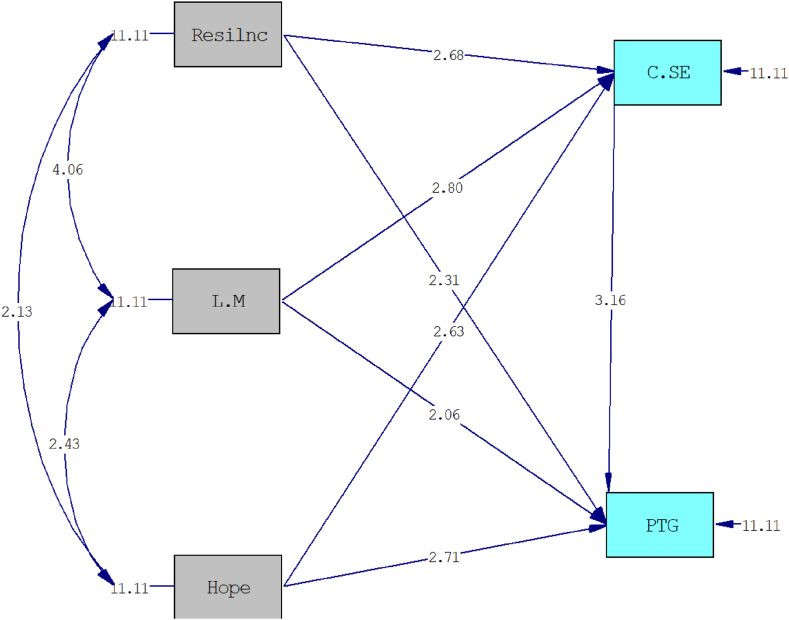
t values the relationship between variables. Note: Resilnc: Resiliance; L.M: Life Meaning; C.SE: Coping Self-Efficacy; PTG: Post-traumatic Growth.

Fig. 3 displays the standard parameters for the paths in the model, while Fig. 4 shows the corresponding t values for these paths.

## 4 Discussion

The purpose of this research was to predict the PTG among nurses working in the COVID-19 ward by considering hope, the meaning of life, and resilience, while also examining the mediating role of coping self-efficacy. The findings of the study demonstrated that hope, the meaning of life, and resilience directly and significantly influenced PTG in nurses working in the COVID-19 ward. Additionally, the quality of life of these nurses was indirectly affected by hope, the meaning of life, and resilience through coping self-efficacy.

The first hypothesis of this study, i.e., the association between resilience and PTG in nurses is mediated by coping self-efficacy, was confirmed. This finding is consistent with Finest et al.’s study (2021). According to their research, PTG can be accelerated by having high levels of resilience and effective coping mechanisms [48]. Resilience also improves nurses' job satisfaction and strong motivation to overcome obstacles in life, while decreasing job burnout and stress [49,50]. Resilience also improves self-efficacy [51]. Markones (2010) discovered that those with higher levels of self-efficacy are better at managing and overcoming difficult situations [52]. Thus, it is evident that those with high levels of self-efficacy do not experience helplessness in challenging circumstances, suppress negative thoughts, and prevail in challenging circumstances. Since nurses deal with various negative and stressful experiences in their work environment, those with a positive orientation and optimistic attitude are more likely to have a positive interpretation of the traumatic event and are more likely to employ adaptive coping mechanisms [53]. These nurses exhibit greater involvement in patient treatment and care; therefore, they perceive a higher quality of life in their social and professional environments [54]. Positive coping skills training has been associated with PTG, according to Hooper et al. (2018) and Mattson et al. (2018) [55,56].

The second hypothesis of this study suggests that there is a connection between hope and PTG in nurses, and this connection can be influenced by coping self-efficacy. This has been confirmed and supported by Azim and Kiran (2022). According to the findings of their study, PTG and self-efficacy are positively correlated [57]. Additionally, Di Corrado et al. (2022) revealed that protective elements, such as hope and resilience can be crucial in creating an intervention approach to enhance and strengthen adults' mental health and PTG throughout the COVID-19 pandemic [58]. Hope encourages a feeling of management in nurses that enables them to set goals, as well as, the capacity to create plans to accomplish those goals, as well as the application of adaption strategies and crisis management techniques [59]. It indicates that dealing with a stressful circumstance is better done in the presence of hope since the likelihood of getting a good result is improved. The expectation of positive results improves performance in critical situations. People with high levels of hope may consider stressful situations more challenging which helps them grow [23]. The nurses with help may better deal with stressful situations and improve their self-efficacy [24] which helps them grow after trauma [60].

The third hypothesis of this study states that coping self-efficacy plays a mediating role between the PTG of nurses and the meaning of life. This hypothesis was confirmed, aligning with the findings of Blackburn and P. Owens' study (2015). According to this study, self-efficacy and meaning of life are beneficial in lessening the stress of the veteran [61]. Having meaning in life makes people feel optimistic about themselves and the future and makes them happy with who they are [29]. Pano et al. (2022) found that since the COVID-19 pandemic is a chronically stressful situation, particularly for those employed in the healthcare industry, the meaning of life enables them to manage their stress [62]. Also, the sense of meaning helps people to consider their lives purposeful, to deal with the issues of this stressful profession, and to appreciate the significance of their efforts as something extraordinary [35]. Self-efficacy is also influenced by the sense of meaning in life. Individuals with high self-efficacy are more stable and able to control their thoughts which predicts PTG and leads to PTG [63].

This study, like others, has limitations. For instance, the results cannot be used to establish cause-and-effect relationships because the study's design is correlational. Additionally, it's important to be cautious when generalizing the results since the study was carried out among nurses in Zanjan City. Based on the findings of this study, it is suggested that future research should involve a larger sample size and consider additional psychological variables that may affect PTG. Furthermore, it is recommended to conduct experimental studies using random sampling to determine the causal relationship between the variables in the current research and other factors.

## 5 Conclusion

This study revealed a significant relationship between PTG and resilience, hope, and the meaning of life among Iranian nurses. The current study, in particular, is one of the pioneering investigations into the mediation function of coping self-efficacy in the relationship between resilience, hope, and the meaning of life with PTG in nurses. The study's findings also demonstrate that resilience, hope, and meaning of life, with the mediating role of coping self-efficacy, can all be used to predict PTG in nurses.

## CRediT authorship contribution statement

**Majid Yousefi Afrashteh:** Writing – review & editing, Validation, Methodology, Conceptualization. **Masoumeh Moradi:** Software, Resources. **Leila Rahmandel:** Writing – original draft, Investigation.

## Ethics approval and consent to participate

The present research received approval from the ethics committee of the University of Zanjan with code IR.ZNU.REC.1401.031 before its implementation. Throughout the research, ethical considerations such as confidentiality, honesty, and the participants' right to consent were upheld. At the outset of the study, the research objectives were communicated to the participants, and they were asked to complete an informed consent form if they agreed to participate. They were also informed that they could withdraw from the study at any time. All procedures involving human participants in the study adhered to the ethical standards of the National Research Committee. Individuals completed an informed consent document before participating in the survey and had the right to withdraw from the study at any time.

## Consent for publication

Not applicable.

## Availability of data and materials

The datasets during and/or analyzed during the current study are available from the corresponding author upon reasonable request.

## Funding

The costs related to the implementation of this research were funded by the 10.13039/501100008854University of Zanjan.

## Declaration of competing interest

The authors declare that they have no known competing financial interests or personal relationships that could have appeared to influence the work reported in this paper.
